# PTEN and GATA3 as Key Molecular Mediators Linking Diabetes Mellitus to Osteoarthritis: A Comprehensive Mendelian Randomization, Bioinformatics, and Experimental Study

**DOI:** 10.1155/ije/2838739

**Published:** 2025-12-12

**Authors:** Jian Ding, Xuqiang Liu, Jun Zhang, Zhiping Zhang, Xiaofeng Li

**Affiliations:** ^1^ Department of Orthopedics, The First Hospital of Nanchang, Nanchang, Jiangxi, 33000, China; ^2^ Department of Orthopedics, The First Affiliated Hospital, Jiangxi Medical College, Nanchang University, Nanchang, Jiangxi, 330000, China, ncu.edu.cn; ^3^ Department of Orthopedics, The Third Affiliated Hospital, Jiangxi Medical College, Nanchang University, Nanchang, Jiangxi, 330000, China, ncu.edu.cn; ^4^ Artificial Joints Engineering and Technology Research Center of Jiangxi Province, Nanchang, 330000, China

**Keywords:** bioinformatics, diabetes mellitus, Mendelian randomization, osteoarthritis

## Abstract

**Background:**

Epidemiological studies suggest a potential link between diabetes mellitus (DM) and osteoarthritis (OA), but the molecular mechanisms underlying this association remain unclear. Identifying these mechanisms is crucial for developing targeted therapies for diabetic patients with OA.

**Methods:**

A two‐sample Mendelian randomization approach was used to assess causal relationships between DM and OA. Differential expression analysis of the GSE51588 and GSE21340 datasets identified common differentially expressed genes (co‐DEGs), followed by GO and KEGG enrichment analysis. Protein–protein interaction (PPI) networks were constructed using STRING and Cytoscape, and potential biomarkers were identified via CytoHubba and ROC curve analysis. Transcription factor (TF)–mRNA and mRNA–miRNA regulatory networks were developed to identify potential drug targets through DGIdb. Molecular docking and artificial intelligence (AI)–based ADMET analysis were performed to validate the interaction between GATA3 and PTEN. RT‐qPCR was conducted to confirm the expression of PTEN and GATA3.

**Results:**

Mendelian randomization identified a causal relationship between diabetes‐related SNPs and OA. A total of 142 co‐DEGs were identified, with PTEN and GATA3 showing significant diagnostic relevance. Molecular docking indicated that GATA3 inhibitors exhibited higher binding affinities than PTEN inhibitors, with ZK‐806711 emerging as a promising dual‐target inhibitor. ADMET analysis suggested that Genz‐10850 is suitable for CNS‐targeted therapy. In chondrocytes, hyperglycemia upregulated PTEN and downregulated GATA3 expression.

**Conclusion:**

In conclusion, we identified the molecular mechanisms linking DM and OA, highlighting PTEN and GATA3 as potential therapeutic targets for intervention.

## 1. Introduction

Osteoarthritis (OA) is a condition characterized by the degeneration of articular cartilage and inflammation of the synovial membrane. It is a significant contributor to pain and functional disability among older adults, affecting approximately 16% of the global population [1]. The incidence of OA is increasing due to an aging population, rising obesity rates and more joint injuries. It is estimated that 250 million people currently suffer from symptomatic OA, and this number is expected to rise to 400 million by 2030 [2]. Consequently, it is imperative to develop interventions and treatment strategies to prevent OA occurrence and progression.

Diabetes mellitus (DM) is a metabolic condition characterized primarily by pancreatic beta cell dysfunction and insulin resistance (IR) and is a major risk factor for multiple diseases [3]. The prevalence of DM is approximately 11.6%, with approximately 536.6 million people currently affected by the condition. It is predicted that the number of people living with DM will increase to 592 million by 2035 [4]. The financial burden of diabetes‐related healthcare costs per capita has increased from an estimated $10,179 in 2012 to $12,022 in 2022 [4]. For these reasons, the effective prevention and treatment of DM are critical public health priorities that require urgent attention and resolution.

Both DM and OA are common age‐related diseases associated with metabolic syndromes such as overweight and obesity. Recent studies have shown that, despite their differing clinical manifestations and etiologies, these two diseases are closely related. A recent study investigated the prevalence of OA in patients with type 2 diabetes was 32.65%, which was significantly greater in females (38.05%) than in males (27.41%) and increased with age [5]. Elevated blood glucose levels and diabetes are also independent risk factors for OA, and a high‐glucose environment may lead to joint injury by affecting chondrocyte metabolism [6]. Age, sex, race, obesity, hypertension, and dyslipidemia are known risk factors for DM and OA [7, 8]. However, after considering these risk factors, the relevance between DM and OA remains controversial.

In this study, the potential association between OA and DM was investigated using the Mendelian randomization (MR) method. Integrated bioinformatics was used to identify co‐DEGs and hub genes. Functional annotation, PPI network, mRNA‐microRNA regulatory network, transcription factor (TF) gene regulatory network, and gene–drug association network were constructed based on co‐DEGs and hub genes. The value of the hub genes in the diagnosis of DM and OA was validated using receiver operating characteristic curves and in vitro cellular experiments. It is anticipated that the findings of this study will facilitate the identification of potential early warning markers and therapeutic targets for future research, while also offering new insights into the biological relationship between DM and OA. It is expected that the findings of this study will facilitate the identification of potential early warning biomarkers and therapeutic targets for future research, while also providing new insights into the biological relationship between DM and OA.

## 2. Materials and Methods

### 2.1. GWAS Data Acquisition and Analysis

This study adhered strictly to the three core assumptions of MR analysis: the instrumental variables (IVs) should exhibit a strong correlation with the exposure under investigation, they must not be associated with any confounding factors between the exposure and outcome, and only exposures that directly influence the outcome are considered. Data from GWAS for DM and OA were obtained from the IEU OpenGWAS database with the following IDs: ebi‐a‐GCST005413 and ebi‐a‐GCST90038686. The DM dataset included 12,931 cases and 57,196 controls, while the OA dataset comprised 39,515 cases and 445,083 controls. In the GWAS of DM, SNPs that achieved genome‐wide significance (*p* < 5 × 10^8^) were used as IVs for DM. We examined SNPs for independent inheritance (*p* < 0.001) without linkage disequilibrium and ensured a minimum distance of 10 kb between SNPs. A total of 16 SNPs associated with OA were identified. The GWAS datasets referenced in this study are derived from diverse populations. Specifically, the DM dataset predominantly consists of individuals of European ancestry, while the OA dataset includes a mix of ancestries, primarily European, with some representation from other populations. We acknowledge that the potential for heterogeneity exists due to differences in genetic background and environmental factors among these populations. Additionally, we conducted heterogeneity tests, such as Cochran’s Q, and sensitivity analyses, including leave‐one‐out analyses, to assess the robustness of our findings. We ensured that there was no sample overlap between the DM and OA datasets by cross‐referencing participant IDs and assessing study design details.

To test the assumptions of MR, we evaluated the strength of the IVs using the F‐statistic, calculated as F = (R^2^ (n ‐ k ‐ 1))/(k (1 ‐ R^2^)), where R^2^ indicates the proportion of variance in the exposure explained by the IVs, with an F‐statistic greater than 10 considered indicative of strong IVs. Potential confounding factors were assessed using PhenoScanner to ensure that none of the selected SNPs had significant associations with known confounders related to OA and DM. To assess horizontal pleiotropy, we used the MR‐Egger intercept method, with a nonsignificant intercept (*p* > 0.05) indicating no evidence of pleiotropy. Additionally, the MR‐PRESSO global test was employed to identify and correct for any potential outliers. We utilized MR computational models to explore the potential causal link between diabetes and OA through various approaches, including inverse variance weighted (IVW) analysis, MR‐Egger regression, and methods such as the simple median (SM) and weighted median (WME). The SM provides a robust estimate of causal effects, while the WME offers a more nuanced approach that accounts for varying weights of the IVs. IVW was employed as the primary approach for MR analysis, conducted using R language version 4.3.3 and statistical packages such as TwoSampleMR, MR‐PRESSO, and Mendelian Randomization.

### 2.2. GEO Data Download and Preprocessing

The GSE21340 and GSE51588 datasets were obtained from the NCBI GEO Database along with their corresponding annotation information. The GSE21340 dataset contained 15 non‐DM tissue samples and five DM tissue samples, while the GSE51588 dataset contained 10 normal tissue samples and 40 tissue samples affected by OA. The GSE21340 and GSE51588 datasets underwent quantile normalization via the Limma package function “normalizeBetweenArrays” in R. The gene symbols were obtained from the platform’s annotation files and used to convert the probe IDs. In instances where multiple probes corresponded to a single gene, the probe exhibiting the highest expression level was selected as the representative value for gene expression. Genes with multiple probes were eliminated from the data to ensure that each gene corresponded to a single microarray probe.

### 2.3. Identify Differentially Expressed Genes (DEGs)

Differential expression analysis was performed using the Limma R package (version 3.52.4). Thresholds were set as |logFC| > 0.58 and *p* < 0.05. Heatmaps of the top 20 up‐ and downregulated genes were generated with the “pheatmap” package (version 1.0.12). Volcano plots were created using “ggplot2” (version 3.4.2). All parameters were used at default unless otherwise specified.

### 2.4. Gene Enrichment Analysis

Gene Ontology (GO) and Kyoto Encyclopedia of Genes and Genomes (KEGG) pathway enrichment analyses were conducted using DAVID (version 6.8). Visualization of significant terms (*p* < 0.05, count > 1) was performed with the R package “Goplot” (version 1.0.2). Heatmaps for enrichment results were generated using the online platform 微生信 (https://www.bioinformatics.com.cn), which provides comprehensive data visualization tools.

### 2.5. PPI Interoperability Network

Protein–protein interaction (PPI) networks of co‐DEGs were constructed via STRING database version 12.0 (https://string-db.org) with a confidence score threshold of 0.7 (high confidence). Network visualization and hub gene identification were carried out in Cytoscape (version 3.9.1). Definition of hub genes was performed using topological metrics, such as degree, maximum cluster centrality (MCC), bottleneck, maximum neighbor component (MNC), and eccentricity methods in the Cytoscape plugin cytoHubba. Venn diagrams of shared hub genes were created using the R package “VennDiagram” (version 1.7.3).

### 2.6. ROC Curves and AUC Values

The ROC curve and area under the curve (AUC) are commonly used for evaluating the precision of diagnostic tests in forecasting results. In this study, the pROC software package was used to produce ROC curves for the hub genes and calculate their respective AUC values. Subsequently, the ggplot2 software package was used to visualize the obtained outcomes.

### 2.7. mRNA‒miRNA Interaction Network

To predict TFs and miRNAs associated with PTEN and GATA3, we used the NetworkAnalyst database (http://www.networkanalyst.ca). TFs were predicted based on the JASPAR database, and the miRNA–gene network was predicted and constructed via TarBase from the NetworkAnalyst database. The results were visualized in Cytoscape.

### 2.8. Drug–Gene Interactions

The Drug Gene Interaction Database (DGIdb 4.0) (https://dgidb.org/) is an available resource for drug‐sensitive genomes and drug‐targeted and drug–gene interactions. DGIdb was used to identify potentially druggable genes and associated drugs in PTEN and GATA3.

### 2.9. Protein and Ligands Extraction

The three‐dimensional (3D) X‐ray crystallographic structures of GATA3 and PTEN proteins were obtained from the Research Collaboratory for Structural Bioinformatics (RCSB) Protein Data Bank to serve as molecular targets in the present study. Specifically, the GATA3 protein was retrieved under the PDB ID 4HC7, with a resolution of 2.65 Å. The structural refinement statistics included a free R‐value of 0.264, a working R‐value of 0.226, and an observed R‐value of 0.184, with chain A selected for further computational analyses. Similarly, the PTEN protein structure was acquired under the PDB ID 1D5R, with a resolution of 2.10 Å. The associated refinement values consisted of a free R‐value of 0.276 and a working R‐value of 0.224, ensuring high‐quality structural data for molecular docking and other computational studies. To identify potential drug candidates, an extensive library of experimental drug compounds was curated for virtual screening. All ligands were initially downloaded in Structure Data File (SDF) format, followed by a series of preprocessing steps to optimize their chemical structures for docking simulations. Hydrogen atoms were explicitly assigned to each molecular structure at physiological pH 7.4 to ensure proper protonation states. Subsequently, structural energy minimization was performed using the Merck Molecular Force Field (MMFF94) with the conjugate gradient algorithm, employing 1000 iterations to achieve an energetically favorable conformation. Following the optimization process, the ligand structures were converted into the required file formats for docking studies. The Open Babel 3.1.1 software tool was employed to generate the Protein Data Bank, Partial Charge (Q), and Torsion Tree (PDBQT) format, as well as the MOL2 format, ensuring compatibility with molecular docking and simulation platforms. These computational preparations facilitated the accurate prediction of ligand–protein interactions, providing a strong foundation for identifying promising therapeutic candidates.

### 2.10. Molecular Docking

The molecular docking simulations for screening multiple ligands against specific protein targets were conducted using AutoDock Vina, a highly efficient docking algorithm integrated within the PyRx software suite. PyRx is a widely adopted computational drug discovery platform designed to facilitate high‐throughput virtual screening by enabling the rapid assessment of large chemical libraries against biological targets. This integration provides a seamless and streamlined approach for identifying potential drug candidates by evaluating their binding affinities and interaction profiles with target proteins. One of the distinguishing features of PyRx is its ability to perform multiple docking operations concurrently, significantly enhancing computational efficiency compared to traditional docking software. This parallel processing capability enables the simultaneous assessment of numerous ligand–protein complexes, drastically reducing the overall computational time required for large‐scale virtual screening experiments. The incorporation of AutoDock Vina further augments the docking efficiency, as it leverages multithreading technology to accelerate the ligand optimization process and refine binding pose predictions with enhanced accuracy. The synergy between PyRx and AutoDock Vina provides researchers with an intuitive, user‐friendly platform that simplifies the virtual screening workflow while maintaining a high degree of precision in predicting molecular interactions. For this study, an extensive library of experimental drug candidates was curated from PubChem, a publicly available chemical repository, to identify potential therapeutic agents targeting GATA3 and PTEN proteins. Each ligand underwent molecular docking simulations to predict its binding affinity, and the top‐ranked compounds based on their docking scores were shortlisted for subsequent in‐depth analysis. To ensure accurate and meaningful docking results, the active binding sites of the target proteins were meticulously identified, and grid boxes were strategically positioned to encompass the key interaction regions. These structural parameters were utilized to compute binding energy values, allowing for a comprehensive evaluation of molecular interactions between the ligands and target proteins. By employing this systematic in silico approach, the study effectively prioritized lead compounds with favorable binding affinities, paving the way for further refinement through advanced computational techniques and potential experimental validation. The integration of AutoDock Vina within PyRx thus proved to be an invaluable strategy in streamlining the drug discovery pipeline, enabling precise, high‐throughput screening of novel therapeutic agents against critical protein targets.

### 2.11. ADMET Analysis

The absorption, distribution, metabolism, and excretion (ADME) properties of the designed compounds were systematically evaluated using the QikProp module. This computational tool predicts a comprehensive set of physicochemical and pharmacokinetic parameters that are crucial for assessing a compound’s drug‐like behavior. The evaluation encompasses key descriptors such as solubility, permeability, metabolic stability, and other factors influencing bioavailability and therapeutic potential. Furthermore, the toxicity profile of the compounds was predicted using the ADMETlab 2.0 platform (https://admetmesh.scbdd.com/), which provides insights into potential adverse effects, safety concerns, and overall pharmacological viability. To enhance the reliability of the predictions, an additional AI‐based computational approach was employed to reassess and validate the outcomes generated by QikProp (https://admet.ai.greenstonebio.com). Specifically, the top 1 candidate molecules that have significant interaction with GATA3 and PTEN were subjected to an independent re‐evaluation to cross‐verify the predicted pharmacokinetic and toxicity parameters. In addition to ADME and toxicity assessments, all the designed molecules were further analyzed to determine their drug‐likeness and bioavailability. These evaluations were based on established criteria such as Lipinski’s Rule of Five, Veber’s rules, and other computational frameworks that predict oral bioavailability and overall suitability as therapeutic agents. The combined analyses provide a robust foundation for selecting the most promising drug candidates for further preclinical and experimental validation.

### 2.12. Cell Culture

The ATDC5 mouse chondrogenic cell line was cultured in complete DMEM at a temperature of 37°C with a CO_2_ concentration of 5%. The medium was switched to high‐glucose medium (25 mM glucose) 6–24h before the experiments. The experiment consisted of three groups: ATDC5 cells cultured in normal medium (5.5 mM glucose) and ATDC5 cells cultured in a high‐glucose medium with 25 mM glucose for either 6 or 24 h.

### 2.13. RT‐qPCR

RT‐qPCR was used to validate the hub genes results**.** The expression levels of PTEN and GATA3 were measured via RT‐qPCR using total RNA from three sets of ATDC5 cell line samples, as previously described. Subsequently, RT‐qPCR was performed using an ABI7500 Real‐Time PCR System, following the manufacturer’s instructions. The cycling conditions for RT‐qPCR consisted of an initial denaturation step at 95°C for 10 min, followed by 40 cycles of denaturation at 95°C for 10 s, annealing at 58°C for 30 s, and extension at 72°C for 30 s (Table 1).

**Table 1:** Primer sequences utilized in PCR for gene expression analysis.

	Forward (5′‐3′)	Reverse (5′‐3′)
β‐actin	AGGGAAATCGTGCGTGAC	CAT​ACC​CAA​GAA​GGA​AGG​CT
GATA3	TCT​GTC​CGT​TTA​CCC​TCC​G	CGT​CTC​CAG​CTT​CAT​GCT​ATC​T
PTEN	TGA​GAG​ACA​TTA​TGA​CAC​CGC​C	GGA​ATT​GTG​ACT​CCC​TTT​TTG​TC

## 3. Results

### 3.1. Two‐Sampled MR Analysis Results

We employed IVW, MR‒Egger, WM, and MR‐PRESSO methods to analyze the causal relationships. The IVW findings revealed a significant positive link between DM and the susceptibility to OA development (OR = 1.00252, 95% CI: 1.0005543‐1.00449; *p* = 0.012). The analysis of the MR‐Egger intercept did not indicate any evidence of horizontal pleiotropy (*p* = 0.459). MR‐PRESSO did not identify any potential outliers. The MR results remained consistent after leave‐one‐out sensitivity analysis (*p* = 0.012). This study has established the presence of a direct and positive causal association between DM and OA, indicating that DM may be considered as a contributing factor to the development of OA (Figure 1).

Figure 1Mendelian randomization (MR) plots for the association between diabetes and osteoarthritis. (a) Results of the MR analysis between exposure and outcome factors, which showed that the lines of the different algorithms were generally upward sloping, with slopes greater than 0, indicating that diabetes was an adverse factor for the development of osteoarthritis. (b) Heterogeneity analysis of the SNPs, with points to the left and right of the IVW line being approximately symmetrical. (c) Forest plots for the SNPs, with diabetes promoting the development of osteoarthritis. (d) Sensitivity analysis of the leave‐one‐out method to determine the effect of each SNP on the results of the Mendelian randomization analysis, the overall error line did not change much after excluding each SNP.

**Figure:**
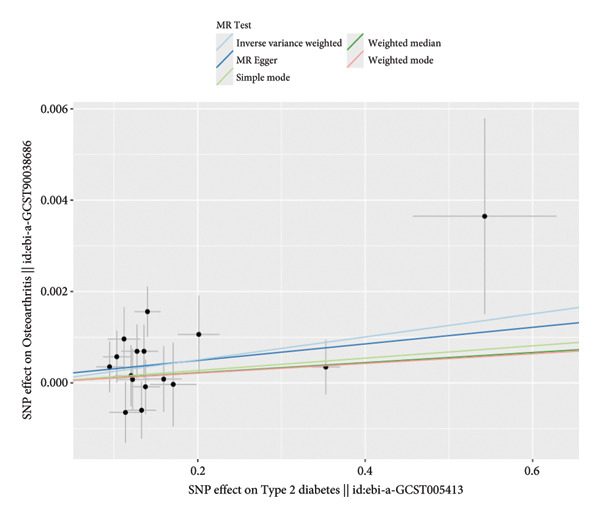
(a)

**Figure:**
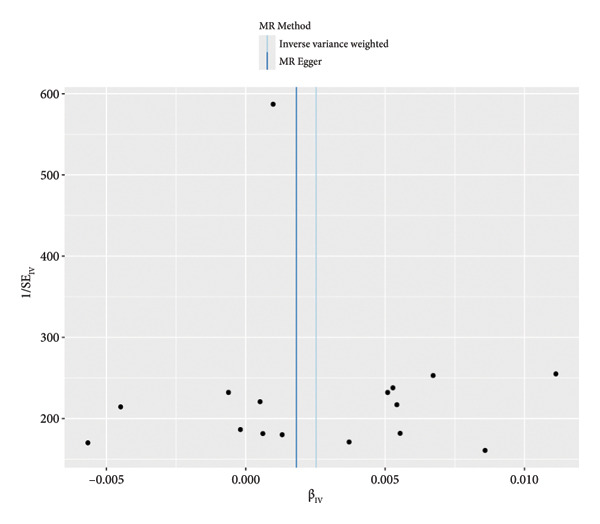
(b)

**Figure:**
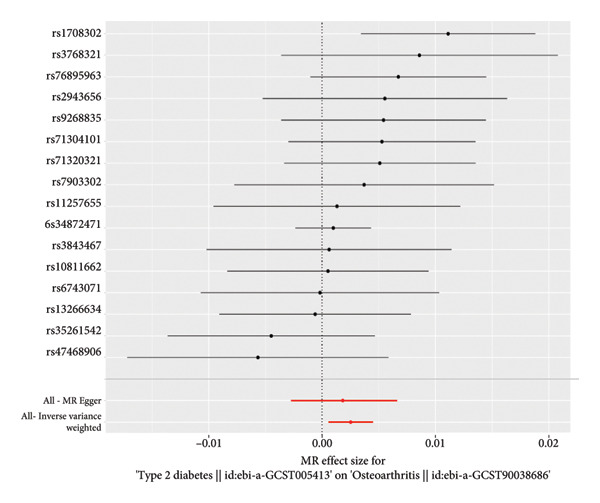
(c)

**Figure:**
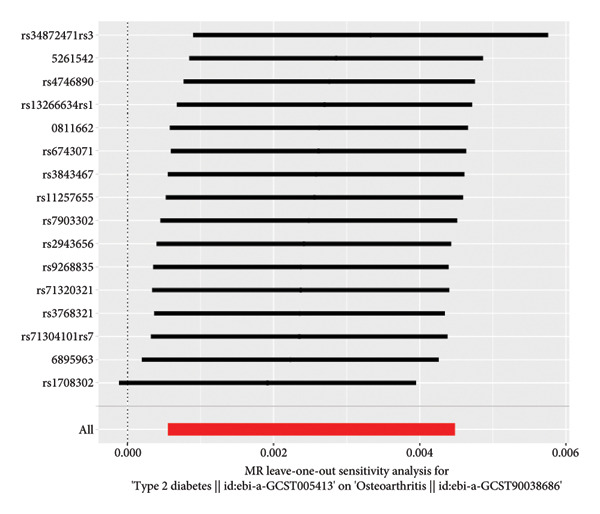
(d)

### 3.2. Identification of the Co‐DEG

A total of 1087 DEGs were screened in the GSE21340 dataset, which included 620 upregulated genes and 467 downregulated genes, using the screening thresholds of *p* < 0.05 and |logFC| > 0.58 (Figure 2(b)). Similarly, the GSE51558 dataset contained 3884 DEGs, with 2012 upregulated genes and 1872 downregulated genes (Figure 2(d)). Heatmaps were generated to display the top 20 genes that showed increased and decreased expression, ranked by logFC in each dataset (Figures 2(a) and 2(c)). By taking the intersection of the DEGs between the two datasets, we obtained 142 common differentially coexpressed genes (co‐DEGs), including 65 upregulated genes (Figure 2(e)) and 77 downregulated genes (Figure 2(f)).

Figure 2Differential gene expression analysis of the diabetes and osteoarthritis datasets. (a) A heatmap for the DEGs of GSE51558 was generated using the pheatmap packages in the R environment (version 4.3.3). (b) The volcano plot of gene expressions in OA(GSE51558). (c) A heatmap for the DEGs of GSE21340. (d) The volcano plot of gene expressions in DM(GSE21340). The red and blue dots indicate upregulated and downregulated DEGs, respectively. (e) Venn diagrams of upregulated DEGs from two datasets. (f) Venn diagrams of downregulated DEGs from two datasets.

**Figure:**
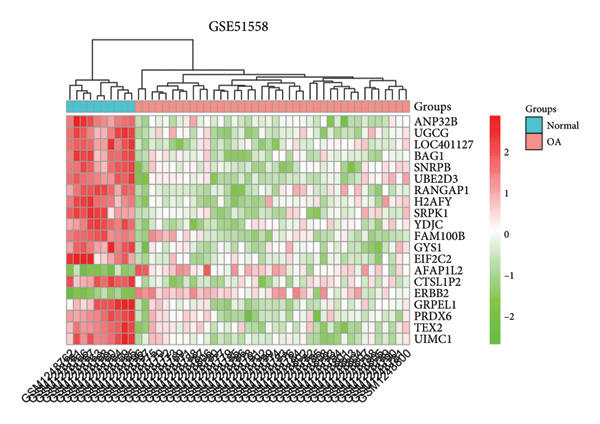
(a)

**Figure:**
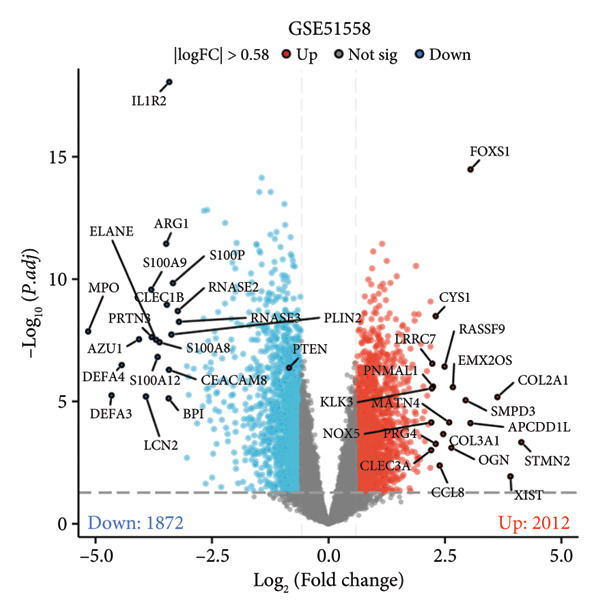
(b)

**Figure:**
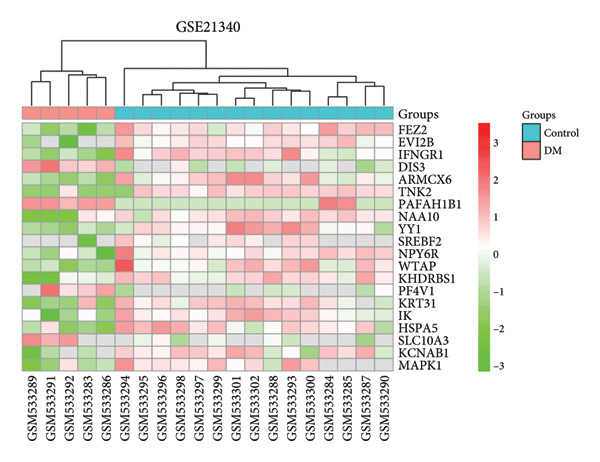
(c)

**Figure:**
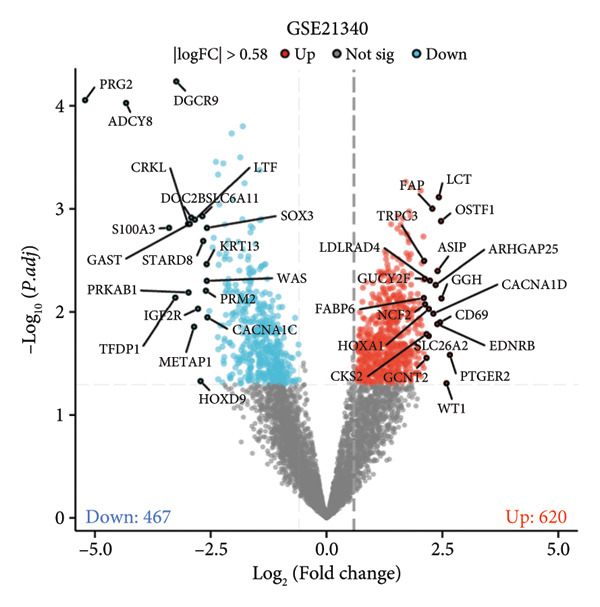
(d)

**Figure:**
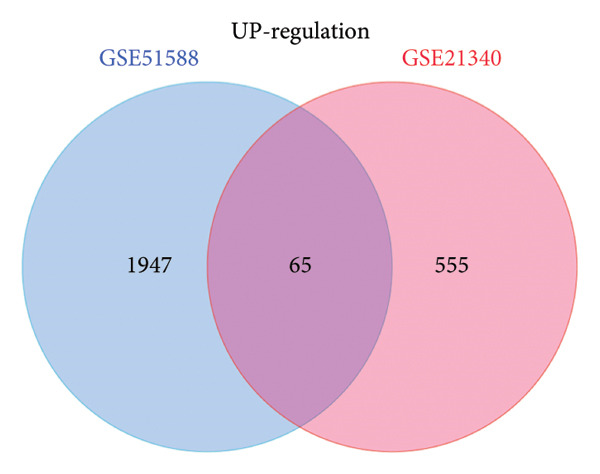
(e)

**Figure:**
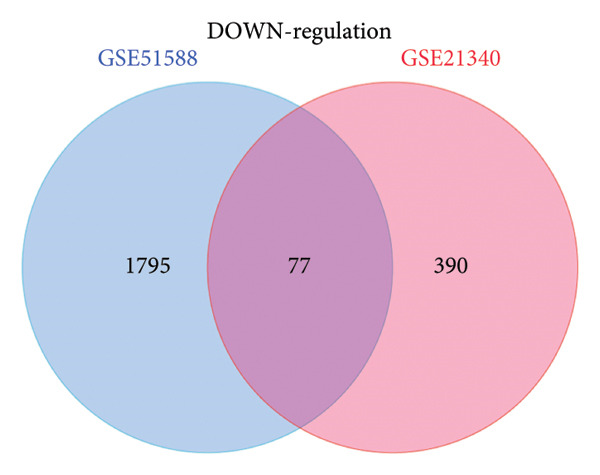
(f)

### 3.3. Functional Enrichment Analysis

The analysis of GO terms revealed a significant concentration of co‐DEGs in functional groups linked to cell adhesion, negative regulation of apoptotic processes, inflammatory responses, positive regulation of cell proliferation, and negative regulation of inflammatory responses to antigenic stimuli (Figure 3(a)). According to the KEGG pathway enrichment analysis, these co‐DEGs exhibited significant enrichment in the cancer, phospholipase D signaling, Fc epsilon RI signaling, PI3K‐Akt signaling, calcium signaling, cAMP signaling, and Rap1 signaling pathways (Figure 3(b)).

Figure 3Functional annotations of the differential genes. (a) GO analysis of 142 co‐DEGs; (b) KEGG analysis of these co‐DEGs.

**Figure:**
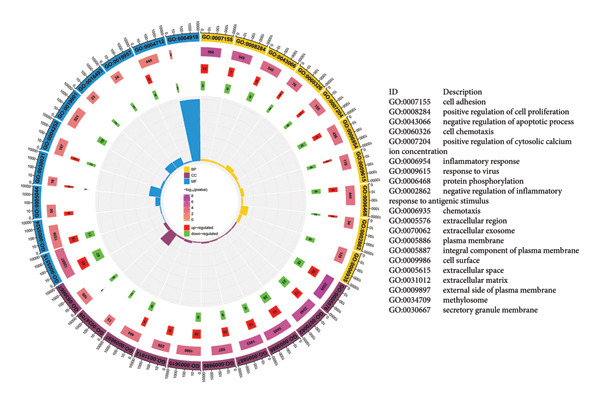
(a)

**Figure:**
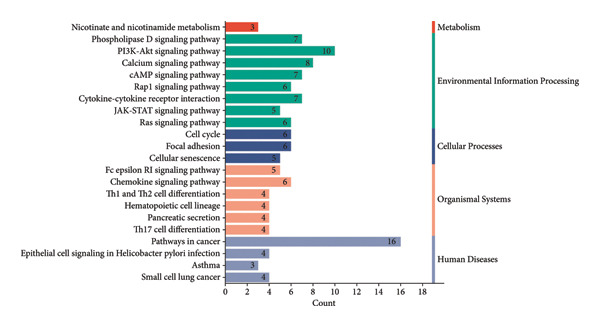
(b)

### 3.4. Protein‒Protein Interactions and Co‐DEGs

The PPI network was constructed by importing the 142 co‐DEGs into the STRING database and displayed via Cytoscape. This network comprised 141 nodes and 255 edges (Figure 4(a)). The 10 most significant gene modules from the PPI network were identified in a sequential manner through the use of five distinct algorithms, namely MCC, degree, MNC, bottleneck, and eccentricity, within the cytoHubba plugin for the Cytoscape software. By examining the overlap between the five genome modules, two key genes were identified as hubs: PTEN and GATA3 (Figure 4(b)).

Figure 4Protein–protein interaction (PPI) network. (a) Protein–protein interaction network of the co‐DEGs. The PPI network contained 141 nodes and 255 edges. (b) Venn diagram illustrating gene overlap which obtained by 5 cytoHubba plug‐in algorithms. (c) Regulatory network of Hub genes contained 23 nodes and 24 edges.

**Figure:**
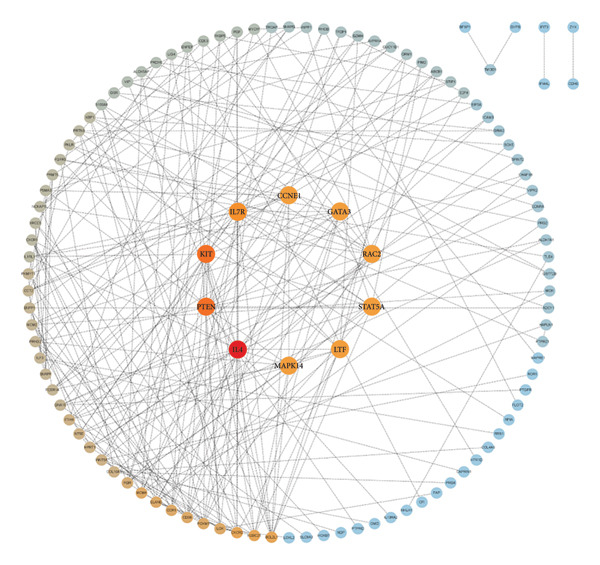
(a)

**Figure:**
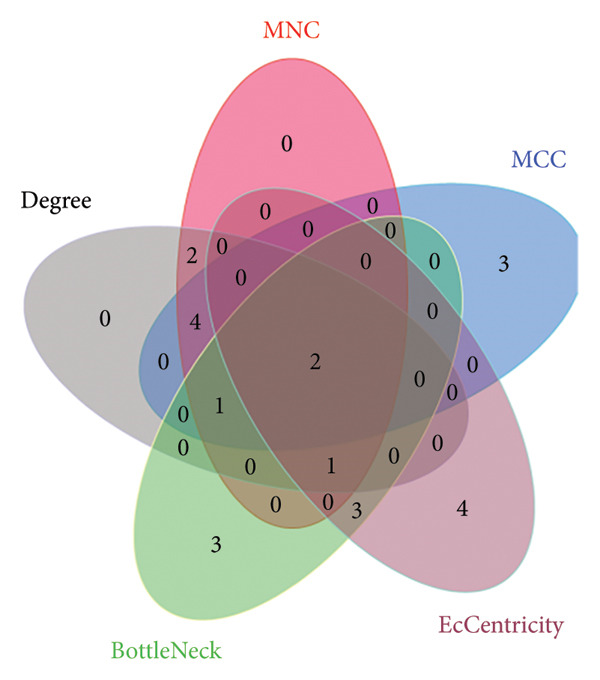
(b)

**Figure:**
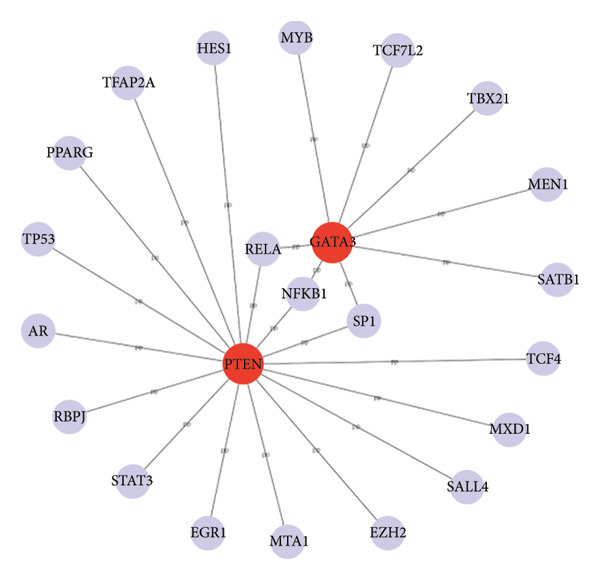
(c)

### 3.5. ROC Curve

Subsequently, the evaluation of the hub genes’ diagnostic efficacy was conducted through the use of ROC curve analysis on the GSE21340 and GSE51588 datasets. ROC curve analysis revealed that in the DM group (i.e., in the GSE21340 dataset), the AUC of PTEN was 0.547, whereas the AUC of GATA3 was 0.853. This indicates that GATA3 had better diagnostic performance. In the OA group (i.e., in the GSE51588 dataset), the AUCs for PTEN and GATA3 were 0.930 and 0.865, respectively, indicating that PTEN had better diagnostic performance (Figure 5).

Figure 5ROC curve analysis for PTEN and GATA3. (a) ROC curves on the GSE21340 dataset. (b) ROC curves on the GSE51558 dataset.

**Figure:**
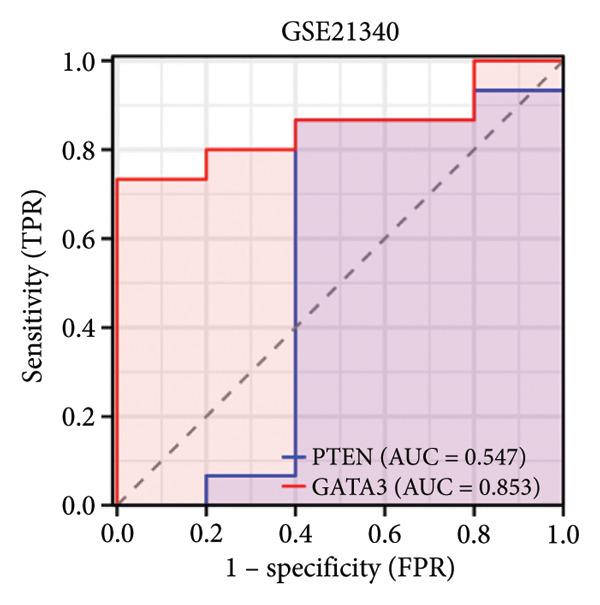
(a)

**Figure:**
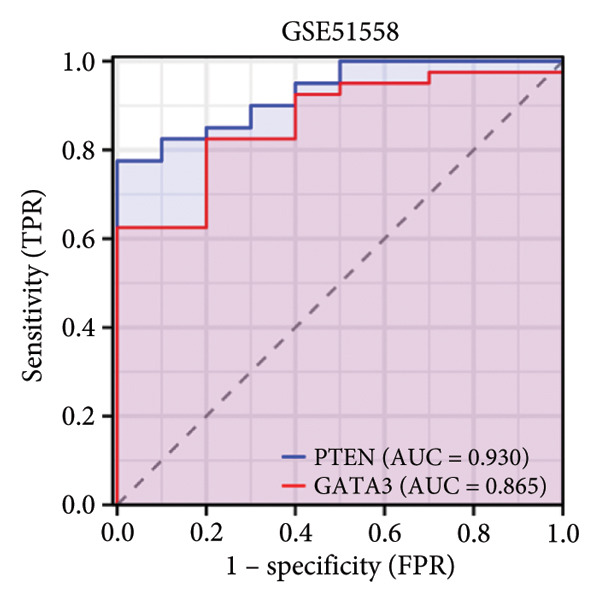
(b)

### 3.6. TF–mRNA Interaction Network

The TF mRNA regulatory network was constructed using the JASPAR database from the NetworkAnalyst website (https://www.networkanalyst.ca). The network consists of 23 nodes and 24 edges, enriched in an abundance of pathways including the PI3K‐Akt and MAPK signaling pathways (Figure 4(c)).

### 3.7. mRNA‒miRNA Regulatory Network

The miRNAs potentially targeting hub genes were predicted using the TarBase v8.0 database and visualized using Cytoscape software. The results revealed 159 nodes and 158 edges, with hsa‐mir‐1‐3p, hsa‐mir‐200‐3p, hsa‐mir‐374a‐5p, and hsa‐mir‐21‐5p presenting the highest connectivity (Figure 6(a)).

Figure 6mRNA‒miRNA regulatory network and potential drug predictions. (a) The regulatory network between miRNAs and hub genes. (b) Gene–drug interaction in DGIdb.

**Figure:**
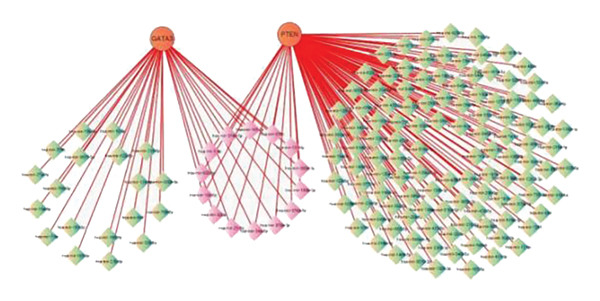
(a)

**Figure:**
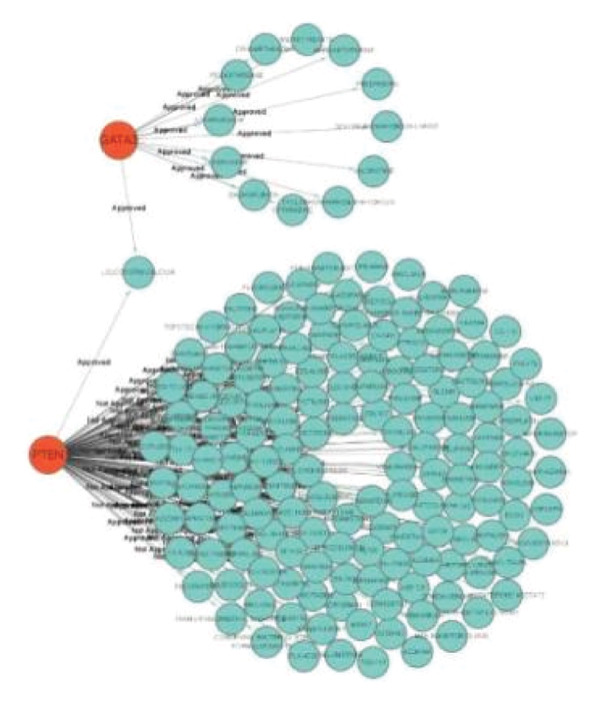
(b)

### 3.8. Potential Drug Predictions

The potential target drugs for the first two hub genes were predicted using the DGIdb database. The results indicate that the two hub genes had 158 potential corresponding therapeutic drugs, 71 of which were approved by regulatory authorities, including metformin, methotrexate, and paclitaxel, suggesting that these drugs may influence the progression of this type of disease (Figure 6(b)).

### 3.9. Molecular Docking

The molecular docking results for PTEN and GATA3 inhibitors reveal key insights into their binding affinities, molecular properties, and potential as therapeutic candidates. PTEN, a tumor suppressor regulating cell growth, was targeted by compounds with binding affinities ranging from −7.7 to −7.1 kcal/mol, while GATA3, a TF involved in immune regulation and cancer progression, exhibited stronger interactions with inhibitors scoring between −8.8 and −8.7 kcal/mol.

Among PTEN inhibitors, Genz‐10850 (−7.7 kcal/mol, MW 393.5 Da) displayed the highest binding affinity, despite having no hydrogen bond donors (HBD = 0) and only one acceptor (HBA = 1).

Other notable inhibitors included 3‐(2‐aminoquinazolin‐6‐yl)‐4‐methyl‐N‐[3‐(trifluoromethyl)phenyl]benzamide (−7.5 kcal/mol, MW 422.4 Da), which had two hydrogen bond donors and five acceptors, suggesting increased stability. ZK‐806711 (−7.5 kcal/mol, MW 455.6 Da) also showed significant potential, possessing six rotatable bonds that could enhance flexibility in binding. Similarly, 4‐[(7‐oxo‐7h‐thiazolo[5,4‐e]indol‐8‐ylmethyl)‐amino]‐n‐pyridin‐2‐yl‐benzenesulfonamide (−7.3 kcal/mol, MW 449.5 Da) exhibited multiple hydrogen bonding interactions (HBD = 2, HBA = 5), which likely contributed to its moderate affinity.

The lowest binding affinity among PTEN inhibitors was observed for 2‐(biphenyl‐4‐sulfonyl)‐1,2,3,4‐tetrahydro‐isoquinoline‐3‐carboxylic acid (−7.1 kcal/mol, MW 393.5 Da), suggesting a relatively weaker interaction. On the other hand, GATA3 inhibitors demonstrated stronger interactions, with N‐cyclopropyl‐4‐methyl‐3‐[1‐(2‐methylphenyl)phthalazin‐6‐yl]benzamide (−8.8 kcal/mol, MW 393.5 Da) and N‐coelenterazine (−8.8 kcal/mol, MW 491.5 Da) showing the highest affinities. N‐Coelenterazine, with its three hydrogen bond donors and acceptors, may form more stable interactions compared to others. JNJ‐27390467 (−8.7 kcal/mol, MW 412.5 Da) and ZK‐806711 (−8.7 kcal/mol, MW 455.6 Da) also exhibited strong affinities, indicating their potential as effective inhibitors.

Notably, ZK‐806711 emerged as a dual‐target inhibitor, binding both PTEN (−7.5 kcal/mol) and GATA3 (−8.7 kcal/mol), which suggests it may have multitarget therapeutic applications.

Additionally, 1‐{(2s,5s)‐4‐fluoro‐5‐[(trityloxy)methyl]tetrahydrofuran‐2‐yl}pyrimidine‐2,4(1h,3h)‐dione (−8.7 kcal/mol, MW 472.5 Da) featured seven rotatable bonds, indicating enhanced flexibility and adaptability in binding. Overall, GATA3 inhibitors exhibited stronger binding affinities compared to PTEN inhibitors, suggesting a greater potential for therapeutic intervention. The role of hydrogen bonding was evident, as seen in N‐coelenterazine and 4‐[(7‐oxo‐7h‐thiazolo[5,4‐e]indol‐8‐ylmethyl)‐amino]‐n‐pyridin‐2‐yl‐benzenesulfonamide, which demonstrated increased stability through higher hydrogen bond interactions. Additionally, molecular weight and flexibility influenced binding efficiency, as larger molecules with multiple rotatable bonds, such as ZK‐806711, showed promising interactions (Table 2). These findings highlight the potential of these compounds as lead candidates, warranting further in vitro and in vivo studies for optimization and validation.

**Table 2:** The molecular docking for targeting PTEN and GATA3.

PTEN								
**ID**	**Name**	**Formula**	Score	MW	**HBD**	**HBA**	**RB**	**NOA**
DB04289	Genz‐10850	C26H23N3O	−7.7	393.5	0	1	3	4
DB06925	3‐(2‐Aminoquinazolin‐6‐yl)‐4‐methyl‐N‐[3‐(trifluoromethyl)phenyl]benzamide	C23H17F3N4O	−7.5	422.4	2	3	4	5
DB03373	ZK‐806711	C27H31N6O	−7.5	455.6	1	1	6	7
DB06844	4‐[(7‐Oxo‐7h‐thiazolo[5,4‐e]indol‐8‐ylmethyl)‐amino]‐n‐pyridin‐2‐yl‐benzenesulfonamide	C21H15N5O3S2	−7.3	449.5	2	5	6	8
DB03207	2‐(Biphenyl‐4‐sulfonyl)‐1,2,3,4‐tetrahydro‐isoquinoline‐3‐carboxylic acid	C22H19NO4S	−7.1	393.5	1	4	5	5
**GATA3**								
DB07307	N‐Cyclopropyl‐4‐methyl‐3‐[1‐(2‐methylphenyl)phthalazin‐6‐yl]benzamide	C26H23N3O	−8.8	393.5	1	3	5	4
DB04118	N‐Coelenterazine	C30H25N3O4	−8.8	491.5	3	3	8	7
DB06962	JNJ‐27390467	C26H24N2O3	−8.7	412.5	1	1	4	5
DB04685	1‐{(2s,5s)‐4‐Fluoro‐5‐[(trityloxy)methyl]tetrahydrofuran‐2‐yl}pyrimidine‐2,4(1h,3h)‐dione	C28H25FN2O4	−8.7	472.5	0	2	7	6
DB03373	ZK‐806711	C27H31N6O	−8.7	455.6	1	1	6	7

### 3.10. ADMET

Genz‐10850 and N‐cyclopropyl‐4‐methyl‐3‐[1‐(2‐methylphenyl)phthalazin‐6‐yl]benzamide exhibit notable differences in their pharmacokinetic and physicochemical properties, impacting their potential therapeutic applications (Table 3). Both compounds have nearly identical molecular weights (∼393.5 Da), but N‐cyclopropyl benzamide has a higher LogP value (5.47 vs. 4.7), indicating greater lipophilicity, which may enhance membrane permeability but could also reduce aqueous solubility. The topological polar surface area (TPSA) of Genz‐10850 is 39.34 Å^2^, whereas N‐cyclopropyl benzamide has a higher TPSA of 54.88 Å^2^, suggesting improved solubility but possibly lower membrane permeability. In terms of Lipinski’s rule compliance, N‐cyclopropyl benzamide violates fewer rules (3/5) than Genz‐10850 (4/5), implying better drug‐likeness. Regarding absorption and bioavailability, both drugs exhibit good human intestinal absorption, with N‐cyclopropyl benzamide (82.16%) performing slightly better than Genz‐10850 (74.87%).

**Table 3:** Artificial intelligence ADMET analysis.

	Genz‐10850		N‐cyclopropyl‐4‐methyl‐3‐[1‐(2‐methylphenyl)phthalazin‐6‐yl]benzamide		
**Property**	**Value**	**DrugBank Percentile**	**Value**	**DrugBank Percentile**	**Units**
Molecular weight	393.5	64.09%	393.49	64.09%	Dalton
LogP	4.7	86.04%	5.47	91.39%	Log‐ratio
Hydrogen bond acceptors	2	16.38%	3	31.17%	#
Hydrogen bond donors	1	36.68%	1	36.68%	#
Lipinski rule of 5	4	63.80%	3	20.92%	# of 4
Quantitative estimate of druglikeness (QED)	0.54	51.80%	0.5	46.92%	—
Stereo Centers	0	22.49%	0	22.49%	#
Topological polar surface area (TPSA)	39.34	22.14%	54.88	34.24%	Å2
Human intestinal absorption	1	74.87%	1	82.16%	—
Oral bioavailability	0.74	41.88%	0.93	81.97%	—
Aqueous solubility	−4.89	15.70%	−6.06	5.00%	log(mol/L)
Lipophilicity	4.15	96.51%	4.56	98.53%	Log‐ratio
Hydration free energy	−10.57	40.95%	−8.76	58.78%	kcal/mol
Cell‐effective permeability	−4.98	50.14%	−5.01	48.16%	log(10‐6 cm/s)
PAMPA permeability	0.86	60.68%	0.85	60.14%	—
P‐Glycoprotein inhibition	0.91	94.65%	0.87	91.59%	—
Blood–brain barrier penetration	0.95	76.54%	0.76	52.00%	—
Plasma protein binding rate	100	92.98%	100	95.08%	%
Volume of distribution at steady state	0	17.33%	0	19.54%	L/kg
CYP1A2 inhibition	0.85	93.41%	0.58	85.50%	—
CYP2C19 inhibition	0.84	95.35%	0.63	88.72%	—
CYP2C9 inhibition	0.8	96.70%	0.61	92.94%	—
CYP2D6 inhibition	0.42	83.64%	0.05	50.33%	—
CYP3A4 inhibition	0.81	91.04%	0.77	89.76%	—
CYP2C9 substrate	0.6	96.67%	0.64	97.79%	—
CYP2D6 substrate	0.62	91.12%	0.26	71.42%	—
CYP3A4 substrate	0.7	72.82%	0.7	73.05%	—
Half‐life	1.08	39.05%	78.27	93.21%	hr
Drug clearance (Hepatocyte)	42.74	55.99%	29.19	43.12%	uL/min/106 cells
Drug clearance (Microsome)	33.68	64.87%	43.07	73.32%	uL/min/mg
hERG blocking	0.96	95.15%	0.72	73.52%	—
Clinical toxicity	0.55	91.70%	0.27	74.14%	—
Mutagenicity	0.34	70.53%	0.46	80.38%	—
Drug‐induced liver injury	0.9	81.62%	0.92	83.99%	—
Carcinogenicity	0.16	51.53%	0.49	88.10%	—
Acute toxicity LD50	3.24	87.51%	2.76	65.06%	log(1/(mol/kg))
Skin Reaction	0.61	67.00%	0.34	39.55%	—
Androgen receptor (full length)	0.01	24.58%	0.02	49.86%	—
Androgen receptor (ligand binding domain)	0.02	61.26%	0.03	77.55%	—
Aryl hydrocarbon receptor	0.47	92.17%	0.42	91.04%	—
Aromatase	0.19	84.76%	0.21	86.74%	—
Estrogen receptor (full length)	0.11	60.26%	0.24	84.06%	—
Estrogen receptor (ligand binding domain)	0.03	66.03%	0.06	80.81%	—
Peroxisome proliferator‐activated receptor gamma	0.04	80.69%	0.1	91.35%	—
Nuclear factor (erythroid‐derived 2)‐like 2/antioxidant responsive element	0.6	88.79%	0.74	94.15%	—
ATPase family AAA domain‐containing protein 5 (ATAD5)	0.06	81.35%	0.31	96.94%	—
Heat shock factor response element	0.18	91.16%	0.26	93.83%	—
Mitochondrial membrane potential	0.63	90.03%	0.75	93.80%	—
Tumor protein p53	0.15	80.69%	0.21	86.00%	—

Additionally, N‐cyclopropyl benzamide has significantly higher oral bioavailability (0.93) than Genz‐10850 (0.74), indicating greater systemic availability upon oral administration. However, Genz‐10850 shows better aqueous solubility (−4.89 log(mol/L) versus −6.06 log(mol/L)), which may facilitate dissolution and absorption in physiological conditions. In terms of distribution, Genz‐10850 demonstrates a higher potential for blood–brain barrier (BBB) penetration (0.95) compared to N‐cyclopropyl benzamide (0.76), making it a more suitable candidate for CNS‐targeted therapies. Both drugs exhibit high plasma protein binding (∼100%), which can impact free drug concentration and pharmacodynamic effects. Their volume of distribution (Vdss) values suggests limited tissue distribution. Metabolism analysis reveals that both compounds strongly inhibit CYP1A2, CYP2C19, CYP2C9, and CYP3A4, raising concerns about potential drug–drug interactions. Genz‐10850 shows higher CYP2D6 inhibition (0.42 vs. 0.05), further increasing the risk of interactions with drugs metabolized by this enzyme. Both compounds serve as substrates for CYP2C9 and CYP3A4, suggesting similar metabolic pathways.

Excretion parameters indicate that N‐cyclopropyl benzamide has a significantly longer half‐life (78.27 h) than Genz‐10850 (1.08 h), implying prolonged systemic circulation and potentially reduced dosing frequency. Hepatocyte clearance for Genz‐10850 is higher (42.74 μL/min/10^6 cells) compared to N‐cyclopropyl benzamide (29.19), whereas the latter exhibits higher microsomal clearance (43.07 vs. 33.68 μL/min/mg), suggesting different elimination pathways. Toxicity analysis highlights key differences between the two compounds.

Genz‐10850 presents a significantly higher hERG blocking potential (0.96 vs. 0.72), indicating a greater risk of cardiotoxicity. Its clinical toxicity score (0.55) is also higher than that of N‐cyclopropyl benzamide (0.27). However, N‐cyclopropyl benzamide shows a greater carcinogenicity risk (0.49 vs. 0.16), which could raise long‐term safety concerns. Additionally, mutagenicity is slightly higher for N‐cyclopropyl benzamide (0.46 vs. 0.34). Overall, Genz‐10850 appears to be more favorable for CNS‐targeted applications due to its higher BBB penetration, better solubility, and shorter half‐life, allowing for quicker elimination. In contrast, N‐cyclopropyl benzamide exhibits superior oral bioavailability and an extended half‐life, which could support prolonged therapeutic effects but raises concerns regarding solubility, carcinogenicity, and metabolic interactions. Both compounds require careful consideration of their ADMET profiles to determine their suitability for clinical development, as predicted in Figure 7.

Figure 7(a) Genz‐10850 and (b) N‐cyclopropyl‐4‐methyl‐3‐[1‐(2‐methylphenyl)phthalazin‐6‐yl]benzamide. Comparison of the clinical toxicity and human intestinal absorption probabilities for a DrugBank reference molecule and an input molecule. Panel A and Panel B display the respective probabilities for each molecule, with clinical toxicity ranging from 0.0 to 1.0 and human intestinal absorption also ranging from 0.0 to 1.0. The comparison highlights differences in toxicity and absorption profiles between the two molecules.

**Figure:**
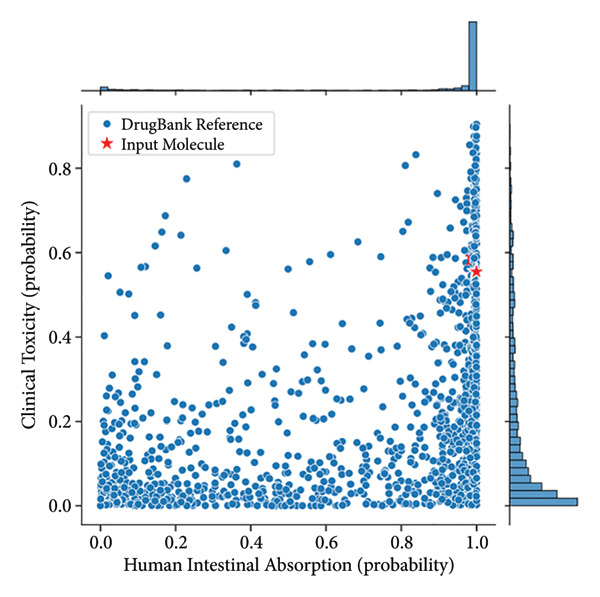
(a)

**Figure:**
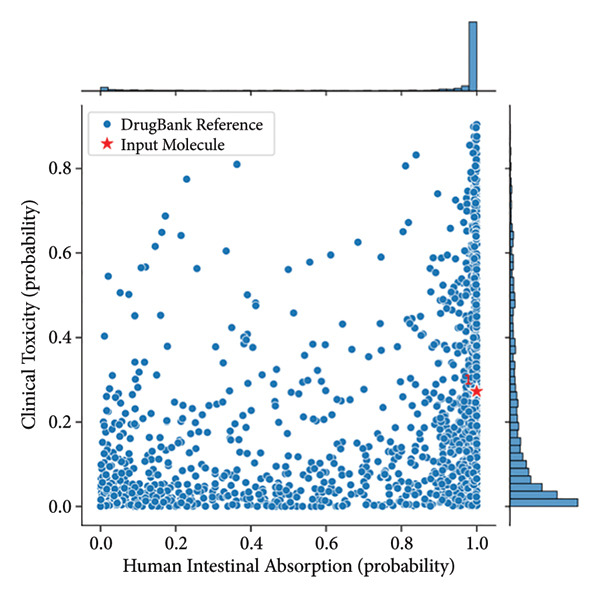
(b)

### 3.11. Validation of PTEN and GATA3

The expression levels of PTEN and GATA3 in ATDC5 cells cultured in a high‐glucose medium were verified using RT‐qPCR. These results showed that under high‐glucose conditions, the mRNA expression level of PTEN was significantly greater in ATDC5 cells than in normal control cells (Figure 8(a), *p* < 0.05). In contrast, the mRNA expression level of GATA3 significantly decreased (*p* < 0.05) (Figure 8(b)). These results indicate that the impact of a high‐glucose environment on ATDC5 cells could be modulated by PTEN and GATA3.

Figure 8PTEN and GATA3 expression in ATDC5 cells cultured in high‐glucose medium. (a) PCR of PTEN mRNA levels in each group. (b) PCR of GATA3 mRNA levels in each group. One asterisk was used for *p* < 0.05, and four asterisks for *p* < 0.0001.

**Figure:**
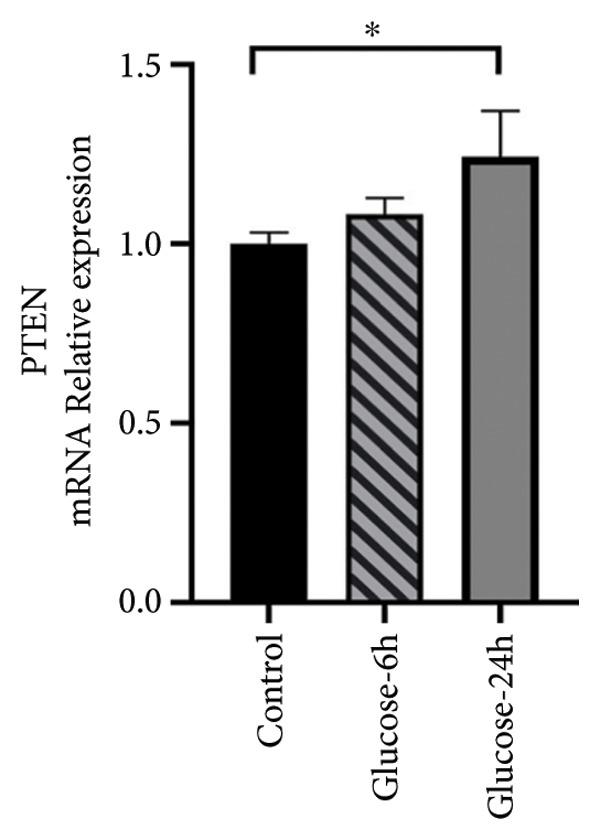
(a)

**Figure:**
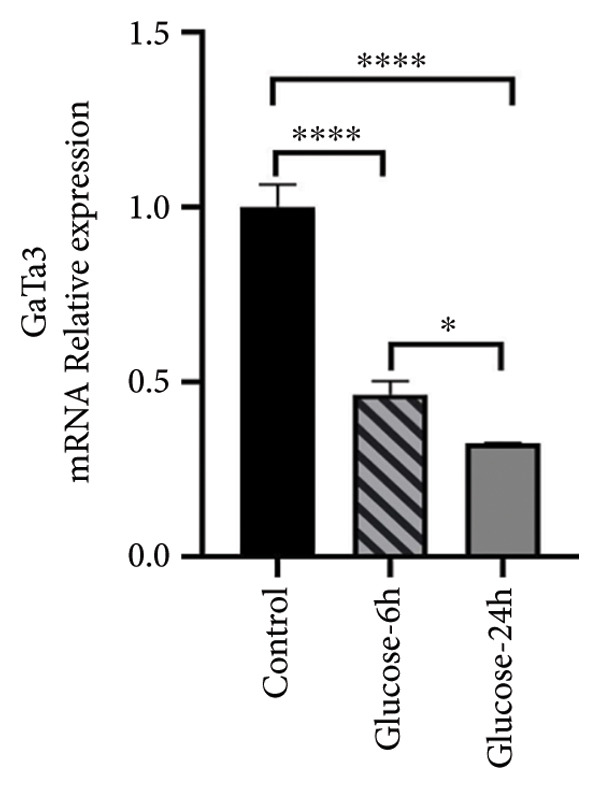
(b)

## 4. Discussion

The association between OA and DM has recently received significant attention from the medical community. OA is associated with elevated fasting glucose (FG) levels [9]. It is also common in diabetes [10]. DM facilitates the progression and development of OA [11, 12]. Chronic diabetes and poor glycemic control are possible elements of risk for symptomatic knee OA [13]. There has been a lack of consensus on the potential correlation between DM and an elevated risk of OA [14]. To date, the relationship between OA and DM remains unclear. Understanding the comorbid mechanisms of DM and OA is crucial for early identification and intervention of OA and DM.

In our study, we analyzed the potential link between OA and DM through two‐sample Mendelian randomization analysis. The results indicated that the presence of DM was a contributing factor in the progression of OA, aligning with previous research outcomes [11, 12]. However, a large‐scale MR study reported no statistically significant causal effects of genetically increased T2D risk, FG, or 2‐h glucose (2hGlu) on either hip or knee OA. These findings suggest that while diabetes may not directly cause OA from a genetic standpoint, the relationship could be mediated by complex metabolic or inflammatory factors rather than by direct genetic predisposition to hyperglycemia [15]. Interestingly, another MR study by Xing et al. reported a complementary causal direction, demonstrating that genetic susceptibility to knee osteoarthritis (KOA), but not hip osteoarthritis (HOA), was associated with an increased risk of type 2 diabetes mellitus (T2DM). These findings suggest a bidirectional relationship between KOA and T2DM, in which not only diabetes predisposes to OA, but OA—particularly of the knee joint—may contribute to the pathogenesis of T2DM through shared metabolic and inflammatory pathways [16].

We analyzed the DM and OA datasets using bioinformatics methods and identified 142 co‐DEGs in DM and OA. The findings indicated the co‐DEGs primarily participated in various cellular functions, including cell adhesion, immune response, apoptosis, and inflammatory response. Additionally, the KEGG pathway analysis revealed that these co‐DEGs presented significant enrichment signaling pathways such as cAMP, PI3K‐Akt, and Rap1. Patients with DM–OA present inhibition of the PI3K‐Akt signaling pathway [14]. This inhibition results in disruption of the glucose homeostasis and lipid metabolism [17], which in turn leads to the development of IR and the progression of T2DM [18]. In addition, Rap1 signaling pathway is closely linked to chondrocyte apoptosis and the cAMP signaling pathway is linked to inflammation and chondrocyte apoptosis [18, 19]. The involvement of these pathways provides biological plausibility for the observed epidemiological and genetic links. Specifically, the dysregulation of PI3K‐Akt and Rap1 signaling may underlie both chondrocyte apoptosis in OA and impaired insulin signaling in T2DM, potentially explaining why KOA, but not HOA, was genetically linked to diabetes risk.

Taken together, our data and the existing literature suggest a mechanistic framework in which hyperglycemia and IR in DM disrupt the PI3K‐Akt and Rap1 pathways, leading to impaired chondrocyte survival and matrix synthesis. Simultaneously, cAMP pathway dysregulation enhances inflammatory cytokine release and oxidative stress, amplifying chondrocyte apoptosis. The convergence of these pathways promotes a proinflammatory and proapoptotic microenvironment in joint tissues, thereby accelerating OA progression. Moreover, miRNAs such as hsa‐miR‐21‐5p and hsa‐miR‐200‐3p appear to modulate these signaling cascades, acting as post‐transcriptional regulators that fine‐tune PTEN and GATA3 expression. This integrated model highlights that the DM–OA relationship is driven not by a single pathway but by a network of interlinked metabolic, inflammatory, and apoptotic signals that jointly contribute to disease comorbidity.

Recent studies have shown a connection between diabetes and cell aging, in which inflammation plays a significant role in this phenomenon [19, 20]. In pathological IR, the glycolytic pathway in OA chondrocytes is inhibited. This inhibition results in a rise in glucose levels within the cells and elevation of glucose‐6‐phosphate [21]. Consequently, an inflammatory response is stimulated, which promotes cartilage aging [20]. A recent study has shown that, in OA, older chondrocytes are more prone to secrete inflammatory mediators than younger chondrocytes [19]. Furthermore, chronic inflammation is a significant pathogenic factor in several diabetes patients [22]. In our study, most of the pathways enriched in the comorbidity genes were closely related to inflammation and apoptosis, which may explain the association between DM and OA. The integrated signaling model proposed here therefore suggests that inflammation and apoptosis act as key intersection points connecting metabolic dysfunction in DM with cartilage degradation in OA.

Two hub genes, PTEN and GATA3, were identified using the CytoHubba plugin and five calculation methods (MCC, MNC, degree, bottleneck, and eccentricity). The ROC curve for the hub genes indicated that GATA3 and PTEN have a high diagnostic potential for diabetes and OA. GATA3 is a significant diagnostic marker for both conditions, and PTEN is particularly effective in treating OA.

GATA3, a TF containing zinc finger domains, regulates gene expression by interacting with nucleosomal DNA and promoting chromatin remodeling. It has an essential function in determining cell lineages and the development of multiple cell types during embryonic development [23]. Genetic variance in GATA3 is linked to the risk of diabetes and GATA3 has a significant impact on regulating the growth of articular cartilage [24]. GATA3 has the potential to regulate adipogenesis, making it a possible target for treating T2DM and IR [23]. PTEN is an important intracellular protein that inhibits cell proliferation and promotes apoptosis. It plays a major role in various biological processes, including cell polarity, migration, metabolism, growth, cell cycle progression, and stem cell renewal. Upregulation of PTEN expression is closely related to IR and development of T2DM [25]. The expression of PTEN is significantly greater in chondrocytes affected by OA than in healthy cartilage [26], and it is significantly increased in the muscle tissue of individuals diagnosed with diabetes [27]. The results of our experiment demonstrated that the expression of PTEN was increased and that of GATA3 was decreased in ATDC5 cells cultured in a high‐glucose medium, thus corroborating previous findings [26]. When considered collectively, these findings indicate that GATA3 and PTEN play significant roles in the pathogenesis of DM combined with OA. Nevertheless, further study is necessary to determine the specific mechanisms of action. Within our proposed mechanistic model, PTEN acts as a negative regulator of the PI3K‐Akt pathway, linking IR to chondrocyte apoptosis, while GATA3 may influence inflammatory signaling and adipogenic differentiation. Dysregulation of both genes thus integrates metabolic stress from DM with joint tissue degeneration in OA.

In addition, we investigated the mRNA–miRNA and TF–mRNA interaction networks associated with the hub genes PTEN and GATA3 using the NetworkAnalyst platform. This analysis revealed several key miRNAs, notably hsa‐miR‐21‐5p, which displayed significant connectivity within the regulatory framework linking DM and OA. The intricate interplay between these miRNAs and their target genes highlights a complex regulatory landscape that may influence the pathogenesis of these conditions. Moreover, the TF–mRNA network analysis underscored significant pathways, including the PI3K‐Akt signaling pathway, which is crucial in cellular responses related to both DM and OA. Understanding these regulatory interactions provides valuable insights into the molecular mechanisms underlying the comorbidity of DM and OA. The identification of potential miRNA targets and their regulatory roles open avenues for therapeutic interventions aimed at mitigating the adverse effects of DM on OA progression. Future studies should prioritize experimental validation of these interactions to confirm their biological relevance and therapeutic potential in the context of DM and OA. Collectively, these multilevel interactions between miRNAs, TFs, and signaling pathways reinforce the hypothesis that the DM–OA axis is governed by interconnected molecular networks rather than isolated gene effects.

Several potential miRNAs associated with two key hub genes were identified by TarBase v8.0. The most stable miRNAs, hsa‐mir‐1‐3p, hsa‐mir‐374a‐5p, hsa‐mir‐200‐3p, and hsa‐mir‐21‐5p, showed strong connectivity in the correlation between DM and OA. The expression of hsa‐miR‐1‐3p is markedly elevated in individuals diagnosed with type 1 diabetes. This increase is closely related to changes in genes associated with vascular development and cardiovascular pathology [28]. Furthermore, hsa‐miR‐374a‐5p is implicated in the regulation of various diseases, such as pancreatitis, intestinal tumors, and Parkinson’s disease, indicating its potential as a therapeutic target [29–31]. Recent studies indicate that increasing hsa‐mir‐200‐3p levels can prevent the progression of diabetic retinopathy by obstructing the TGF‐β2/Smad pathway [32]. In addition, hsa‐mir‐21‐5p has a significant impact on the onset and progression of diabetes and it has a significant correlation with various diabetic complications such as diabetic retinopathy, renal fibrosis, diabetic nephropathy, IR, and cardiovascular disease. Hsa‐mir‐21‐5p also presents a major regulatory influence on the skeletal system and bone diseases [33]. The targeted regulation of miR‐21‐5p may represent a promising new opportunity for research in the treatment of complex diseases such as OA and DM.

The DGIdb database was used to predict potential therapeutic drugs for two hub genes, metformin, methotrexate, and paclitaxel. These drugs may play a significant role in the treatment of DM and OA. Metformin, in particular, has demonstrated beneficial effects in managing OA and may be associated with a reduced incidence of the condition [34]. Molecular docking results revealed that PTEN and GATA3 inhibitors exhibit promising binding affinities, with GATA3 inhibitors generally showing stronger interactions compared to PTEN inhibitors. Notably, ZK‐806711 emerged as a dual‐target inhibitor, binding both PTEN and GATA3, suggesting multitarget therapeutic potential. Key factors influencing binding efficiency included hydrogen bonding, molecular weight, and flexibility, as seen in compounds like N‐coelenterazine and Genz‐10850. ADMET analysis highlighted differences in pharmacokinetic properties: Genz‐10850 demonstrated better aqueous solubility and blood–brain barrier penetration, making it suitable for CNS‐targeted therapies, while N‐cyclopropyl‐4‐methyl‐3‐[1‐(2‐methylphenyl)phthalazin‐6‐yl]benzamide exhibited superior oral bioavailability and a longer half‐life, supporting prolonged therapeutic effects but raising concerns about solubility and carcinogenicity. Both compounds showed high plasma protein binding and potential for drug–drug interactions due to CYP enzyme inhibition. These findings underscore the therapeutic potential of these inhibitors while emphasizing the need for further optimization and validation to address pharmacokinetic and toxicity challenges.

## 5. Limitations

This study has several limitations that should be acknowledged. First, although quantile normalization was performed on the datasets, no explicit cross‐batch correction methods, such as ComBat, were applied. This omission may impact the robustness of our findings when integrating data from different platforms. Additionally, the small and unbalanced sample sizes in the GEO datasets—particularly the limited number of DM samples—may reduce statistical power and increase variability in differential expression analysis, potentially leading to bias in gene identification. This limitation may affect the reliability of our findings and their applicability to broader populations. Moreover, multiple‐testing correction for DEGs was not applied, which raises the possibility of false positives among the identified DEGs. Future studies employing false discovery rate (FDR) or Bonferroni correction will be needed to enhance the statistical rigor of gene selection.

The data used in this analysis may not fully represent the broader population, and genetic or environmental variations among different groups could influence the relationship between DM and OA, potentially affecting the outcomes. Moreover, the identification and retention of 16 SNPs as IVs is relatively small, which may reduce the statistical power and robustness of the analyses. The limited number of IVs could potentially influence the precision of the estimates and the generalizability of the findings. Although the selected SNPs met stringent criteria for inclusion, such as achieving genome‐wide significance and being independent from linkage disequilibrium, a larger set of IVs would be beneficial for enhancing the reliability of the Mendelian Randomization analysis.

Additionally, although the analytical methods employed, including IVW, MR‐Egger, WM, and MR‐PRESSO, are robust, they may not fully capture the complexity of the biological interactions between DM and OA. These methods, while effective, might overlook subtle or nonlinear relationships that could be critical to understanding the underlying mechanisms.

Moreover, the identification of 142 coexpressed differentially expressed genes (co‐DEGs) may not encompass all relevant genes involved in the DM–OA interaction. The selection criteria, which rely on specific statistical thresholds, could exclude genes that play significant roles but do not meet these thresholds. This limitation highlights the possibility that some biologically important genes may have been overlooked in the analysis.

While the functional pathway analysis revealed significant pathways, the interpretation of these results is complex. Overlapping pathways and interactions between them may obscure the identification of direct causal mechanisms, making it challenging to draw definitive conclusions about the specific pathways driving the DM–OA relationship. Finally, the validation experiments were conducted exclusively in a mouse chondrogenic cell line (ATDC5). Although this model provides valuable mechanistic insights, it may not fully recapitulate the molecular and physiological processes in human tissues. Therefore, additional validation using human‐derived cells, tissues, or clinical samples is required to confirm the translational relevance of these findings.

Future studies should aim to address these limitations by increasing sample size and balance across datasets, applying rigorous multiple‐testing correction, integrating multicohort analyses, and validating findings in human experimental models. Such efforts will improve the reproducibility, generalizability, and clinical relevance of research investigating the molecular mechanisms linking DM and OA.

## 6. Conclusion and Future Directions

This study employed a two‐sample MR analysis to explore the potential causal association between OA and DM. Transcriptome data from patients with DM and OA were evaluated and DEGs and key hub genes that may contribute to their shared pathophysiology were identified. These hub genes are involved in apoptosis and inflammation, suggesting that the inflammatory response could represent a pivotal factor in the correlation between OA and DM. While these findings provide preliminary insights into the molecular interplay between the two diseases, they should be interpreted cautiously given the limited MR sample size and potential biases in the GWAS datasets.

Future studies with larger, independent cohorts and experimental validation are required to confirm these associations and assess the diagnostic or therapeutic relevance of hub genes such as PTEN and GATA3. Future research should focus on exploring genetic variability through next‐generation sequencing to identify additional variants linked to the DM–OA comorbidity, while delving deeper into the molecular mechanisms of signaling pathways like PI3K‐Akt and cAMP to uncover their roles in disease progression. Longitudinal studies tracking patients over time could reveal risk factors and early biomarkers, enabling earlier interventions. Investigating miRNAs, such as hsa‐miR‐21‐5p and hsa‐miR‐200‐3p, offers potential for targeted therapies, and clinical trials are essential to validate therapeutic interventions like metformin for clinical use. Integrating biochemical, genomic, and clinical approaches will provide a more comprehensive understanding of the DM–OA interaction, paving the way for improved prevention and treatment strategies to enhance patient outcomes.

## Ethics Statement

Our study is based on open‐source data, and ethics approval was obtained from the Nanchang First Hospital Medical Ethics Committee (No. KY2024010).

## Disclosure

All authors read and approved the final manuscript and agreed to be accountable for all aspects of the work, ensuring that questions related to the accuracy or integrity of any part of the work are appropriately investigated and resolved.

## Conflicts of Interest

The authors declare no conflicts of interest.

## Author Contributions

Jian Ding contributed to the acquisition and interpretation of data and drafting of the manuscript. Xuqiang Liu, Jun Zhang, and Zhiping Zhang provided material input and data analysis and assisted in revising the manuscript. Xiaofeng Li supervised experimental design and wrote the manuscript.

## Funding

This research received funding from the Science and Technology Bureau of Nanchang Municipality under Grant no. Hongke Zi (2023) 336.

## Data Availability

The data that support the findings of this study are available upon request from the corresponding author. The data are not publicly available due to privacy or ethical restrictions.
